# A High-Precision Algorithm for DOA Estimation Using a Long-Baseline Array Based on the Hearing Mechanism of the Ormia Ochracea

**DOI:** 10.3390/s22031249

**Published:** 2022-02-07

**Authors:** Jiazhi He, Zhen Huang, Xuefeng Feng

**Affiliations:** 1 School of Aerospace Engineering, Tsinghua University, Beijing 100084, China; uestchjz@163.com (J.H.); xuefeng-feng@foxmail.com (X.F.); 2 Beijing National Research Center for Information Science and Technology, Tsinghua University, Beijing 100084, China; 3 Space Center, Tsinghua University, Beijing 100084, China

**Keywords:** direction of arrival estimation, ormia ochracea, fourth-order cumulants, cramér-rao lower bound

## Abstract

Inspired by the Ormia Ochracea hearing mechanism, a new direction of arrival estimation using multiple antenna arrays has been considered in spatially colored noise fields. This parasitoid insect can locate s cricket’s position accurately using the small distance between its ears, far beyond the standard array with the same aperture. This phenomenon can be understood as a mechanical coupled structure existing between the Ormia ears. The amplitude and phase differences between the received signals are amplified by the mechanical coupling, which is functionally equivalent to a longer baseline. In this paper, we regard this coupled structure as a multi-input multi-output filter, where coupling exists between each pair of array elements. Then, an iterative direction-finding algorithm based on fourth-order cumulants with fully coupled array is presented. In this manner, the orientation of the mainlobe can direct at the incident angle. Hence, the direction-finding accuracy can be improved in all possible incident angles. We derive the Cramér-Rao lower bound for our proposed algorithm and validate its performance based on simulations. Our proposed DOA estimation algorithm is superior to the existing biologically inspired direction-finding and fourth-order cumulants-based estimation algorithms.

## 1. Introduction

Presently, direction of arrival (DOA) estimation has attracted intensive interest in target localization with radar, sonar and microphone systems [1,2,3,4,5,6,7]. A variety of methods have been proposed in the literature, which differ by type of measured parameter. Traditional parameters include the amplitude [8] and phase [9,10] of the signal. To estimate the DOA of multiple signals with the same frequency, the DOA estimation algorithms based on antenna array have attracted significant attention over the past few decades. Ref. [11] proposed a DOA estimation algorithm using the linear subspace, which is called MUSIC. On the other hand, maximum likelihood (ML) estimation may also be used in multiple-signal DOA estimation. With the development of sparse signal representation and compressed sensing, a series of new DOA estimation methods have been proposed [12,13,14,15]. Ref. [12] presented a recursive weighted least-squares algorithm named FOCUSS for DOA estimation. In [13], a sparse recovery method based on $\ell_{1}$-norm minimization is proposed, which can handle closely spaced correlated signals with known numbers. Moreover, a joint sparse recovery strategy solves a similar problem using a mixed $\ell_{2,0}$ norm approximation with fewer snapshots [14]. The relevance vector machine (RVM) is another sparsity-inducing technique based on Bayesian learning [16]. The RVM-based beamforming method introduced in [17] can remove the undesirable effects of signal correlation and limited snapshots, whereas its resolution capability may be even worse than MUSIC in some scenarios. To solve the DOA estimation problem better, an efficient ML method based on a spatially overcomplete array output formulation is proposed [18]. The DOA is estimated by the reconstructed array output using a refined 1-D searching procedure. In recent decades, a promising technique called machine learning has been widely used in the DOA estimation problem as well [19,20,21]. These methods establish training sets with a DOA label first, and then derive a mapping from the array outputs to the DOA with existing machine-learning techniques. It has been shown that these methods can reduce computation complexity and perform comparably with the subspace-based methods. However, in general, the theoretical performance of most existing algorithms merely relies on the aperture of antenna arrays and the number of antenna elements, which is difficult to further improve with a given array.

Recently, a parasitoid tachinid fly called Ormia Ochracea has appealed to researchers due to its accurate localization ability of the field cricket using the cricket’s call. The DOA estimation accuracy of the female Ormia can achieve $2^{\circ }$ using the small distance between its ears [22,23], far beyond the performance of existing algorithms under similar conditions. Experimental research indicates that the remarkable estimation performance of the Ormia’s auditory system arises from a special coupled structure between its ears, which amplifies the interaural difference in intensity and arrival time [24,25].

Inspired by the coupled structure of the Ormia’s auditory system, a biologically inspired small-aperture array for direction-finding has been discussed from acoustic and radio perspectives [26,27,28,29,30]. A biologically inspired miniature silicon condenser microphone diaphragm has been designed in [31], which exhibits good directionality and sensitivity. In the radio application, a two-element coupled array is designed as a circuit connecting the received antennas, which mimic the coupled structure between the Ormia’s ears [32,33,34]. In [33], the parameters of circuit elements were determined by the tradeoffs between phase amplification and its output power level. Furthermore, the coupled structure has been extended to multiple sensors. A mechanical coupled structure for triple sensors was proposed in [35], which connects inputs by springs and dash-pots. In the above research, the coupling is achieved by mechanical or circuit structure. In fact, the coupled structures for multiple-sensor arrays are quite complicated and normally hard to implement, especially when the number of sensors increases. Meanwhile, once the structure is determined, the model parameters are difficult to change, which makes it inflexible in practical application. By contrast, from the signal-processing perspective, the coupled structure can be implemented as a digital filter in [36,37,38]. Literature [36] analyzes the performance of an interferometer using two digitally coupled antennas, and implements a direction-finding prototype to verify the theoretical analysis. A multi-input multi-output filter designed for multiple antennas has also been studied in [37,38]. Unfortunately, the accuracy improvement brought by biologically inspired coupling merely exists within a certain range of DOA, which is in inverse proportion to the aperture of the array [36]. However, most of the existing studies assume, either explicitly or implicitly, that the DOA locates the range of accuracy improvement, and the DOA estimation outside the range is not analyzed. Moreover, the received noises are correlated after biologically inspired coupling. Since the coupling matrix is given before DOA estimation, traditional ML estimation can be applied when the noises are whitened [37,38]. However, the estimation performance degrades when the received noise is an unknown-colored Gaussian, due to the nonidealities of the receiving channels [39]. Since the colored Gaussian noise can be greatly suppressed by the fourth-order cumulants (FOC), the DOA estimation methods based on the FOC have attracted an extensive attention, and have been developed in past decades [40,41]. The modified MUSIC algorithms based on the FOC were presented in [42,43]. To reduce the computation complexity of the FOC matrix, ref. [44] downsized the matrix by substituting the beamforming output for the array output, whereas a joint second- and fourth-order DOA estimation method was proposed in [45]. As for the coherent signals, pre-processing techniques such as forward–backward averaging have been applied to improve estimation accuracy [46]. However, the DOA estimation outside the accuracy improvement range is still unsolved with the existing FOC-based DOA estimation methods. Finally, the existing coupled structure for multiple sensors only considers the coupling between neighboring sensors in the array [37,38], and it requires further research for better performance.

In this paper, we consider biologically inspired direction-finding using the coupled array. In contrast to the existing antenna array coupled by its immediate neighboring elements, we research the coupling between each pair of array elements. The design of this coupled array can improve DOA estimation accuracy even further. Then, we implement the coupled structure as a multi-input multi-output digital filter. As mentioned in our prior research [36], the range of accuracy improvement for biologically inspired direction-finding is in inverse proportion to the aperture of the array; thus it can cover all possible incident angles for the Ormia’s ears, which are extremely compactly spaced compared to the wavelength of the sound. However, as for antenna arrays in practice, to achieve the resolution capability for multiple signals and high estimation accuracy, the aperture cannot be as closely spaced as the Ormia’s. This would restrict the range of accuracy improvement significantly. To expand the range of accuracy improvement, we propose an iterative FOC-based estimation method with fully coupled array (IFOCE-FC). In this method, a phase adjustment strategy based on an iterative scheme is used for incident angles outside the range of accuracy improvement. Then, a FOC-based estimation approach is used to estimate the DOA with the phase-adjusted signals in the presence of correlated received noise. Hence, the IFOCE-FC algorithm can improve the resolution capability and DOA estimation performance for all possible incident angles. The Cramér-Rao lower bound (CRLB) for the proposed algorithm is derived as well. Simulations validate that the proposed algorithm outperforms the existing biologically inspired direction-finding method [37] and FOC-based MUSIC algorithm [42] in the presence of spatially colored noise.

The remainder of this paper is organized as follows: Section 2 reviews the principle of biologically inspired direction-finding using a uniform linear array (ULA) and demonstrates the tradeoffs between array aperture and direction-finding range. Section 3 introduces the fully coupled structure for multiple antennas and the biologically inspired array processing. Then, the iterative FOC-based DOA estimation method with fully coupled array is proposed in Section 4. Section 5 analyzes the theoretical performance of the proposed algorithm. We extend our analysis to the uniform circular antenna array (UCA) in Section 6 as well. In Section 7, using Monte Carlo simulations, we compare the proposed algorithm with the existing estimation method and demonstrate its improvement in the estimation performance. Finally, we provide our conclusions in Section 8.

Please note that the incoming signal is assumed to satisfy the narrowband array assumption, which means the propagation time of the signal across the array is much smaller than the reciprocal of signal bandwidth [47]. The assumption ensures that the difference between received signals are merely brought by the carrier phase.

## 2. Background

### 2.1. Mathematical Model

**Figure 1:**
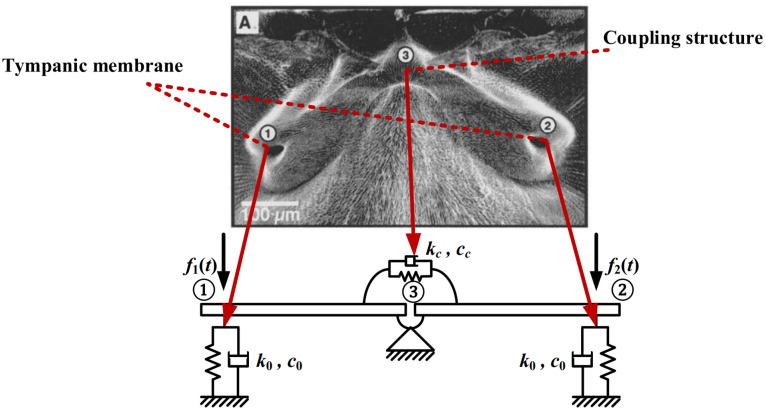
Photograph of the Ormia’s auditory system and its correspondence relationship with the mechanical model. It consists of springs and dash-pots, which reflects the tympanic membrane and the coupled structure.

Biological research in [25] indicates that the outstanding direction-finding performance of the Ormia Ochracea arises from a special mechanical coupled structure in its auditory system, which is shown in Figure 1. This structure consists of springs and dash-pots, with the symmetrical parameters on the bilateral organs. It can be seen that the Ormia’s ears are coupled through the central pivot with rigid bars.

According to the mechanical model mentioned above, the mathematical model of biologically inspired coupled structure can be obtained when the applied forces and springs displacement are changed to the electrical signals [25,37]:

(1) $$\left[\begin{array}{cc} k_{0}+k_{c} & k_{c}\\ k_{c} & k_{0}+k_{c} \end{array}\right]\left[\begin{array}{c} y_{1}\\ y_{2} \end{array}\right]+\left[\begin{array}{cc} c_{0}+c_{c} & c_{c}\\ c_{c} & c_{0}+c_{c} \end{array}\right]\left[\begin{array}{c} \dot{y_{1}}\\ \dot{y_{2}} \end{array}\right]+\left[\begin{array}{cc} m_{0} & \\ \; & m_{0} \end{array}\right]\left[\begin{array}{c} \ddot{y_{1}}\\ \ddot{y_{2}} \end{array}\right]=\left[\begin{array}{c} x_{1}\left(t,\phi \right)\\ x_{2}\left(t,\phi \right) \end{array}\right]$$

where $m_{0}$, $k_{0}$, $k_{c}$, $c_{0}$ and $c_{c}$ account for the effective mass, spring and dash-pot constants of the mechanical model, respectively. The detailed corresponding relation is demonstrated in Figure 1. $x_{i}\left(t,\phi \right),i=1,2$ and $y_{i}\left(t,\phi \right),i=1,2$ are the input and output signals, whereas $\dot{y_{i}}$ and $\ddot{y_{i}},i=1,2$ denote the first and second derivative of the output signals versus time.

We can solve the differential equations and obtain the transfer function by applying the Fourier transform to (1) with zero initial values, as given in [37,38]:

(2) $$\left[\begin{array}{c} Y_{1}\left(j\omega_{c}\right)\\ Y_{2}\left(j\omega_{c}\right) \end{array}\right]=H_{I}\left(j\omega_{c}\right)\left[\begin{array}{c} X_{1}\left(j\omega_{c}\right)\\ X_{2}\left(j\omega_{c}\right) \end{array}\right]=\frac{1}{P(j\omega_{c})}\left[\begin{array}{cc} D(j\omega_{c}) & -N(j\omega_{c})\\ -N(j\omega_{c}) & D(j\omega_{c}) \end{array}\right]\left[\begin{array}{c} X_{1}\left(j\omega_{c}\right)\\ X_{2}\left(j\omega_{c}\right) \end{array}\right]$$

where $\omega_{c}$ is the center frequency of received signals. $D(j\omega_{c})$, $N(j\omega_{c})$ and $P(j\omega_{c})$ can be expressed by the parameters $\left\{m_{0},k_{0},k_{c},c_{0},c_{c}\right\}$ mentioned above:

(3) $$\begin{array}{cc} D(j\omega_{c}) & =-m_{0}\omega_{c}^{2}+\left(c_{0}+c_{c}\right)j\omega_{c}+k_{0}+k_{c}\\ N(j\omega_{c}) & =c_{c}j\omega_{c}+k_{c}\\ P(j\omega_{c}) & =D^{2}\left(j\omega_{c}\right)-N^{2}\left(j\omega_{c}\right) \end{array}$$

The transfer function of the coupled structure can be regarded as a two-input two-output filter system. According to the frequency response of the Ormia’s ears, the coupling amplifies the amplitude and phase differences between its two inputs, accompanied by a sacrificing at the power of output signals. Combining these two factors, the coupled structure can improve the direction-finding accuracy by as much as four times compared to the no-coupling standard array [36].

### 2.2. The Range of Biologically Inspired Direction-Finding

**Figure 2:**
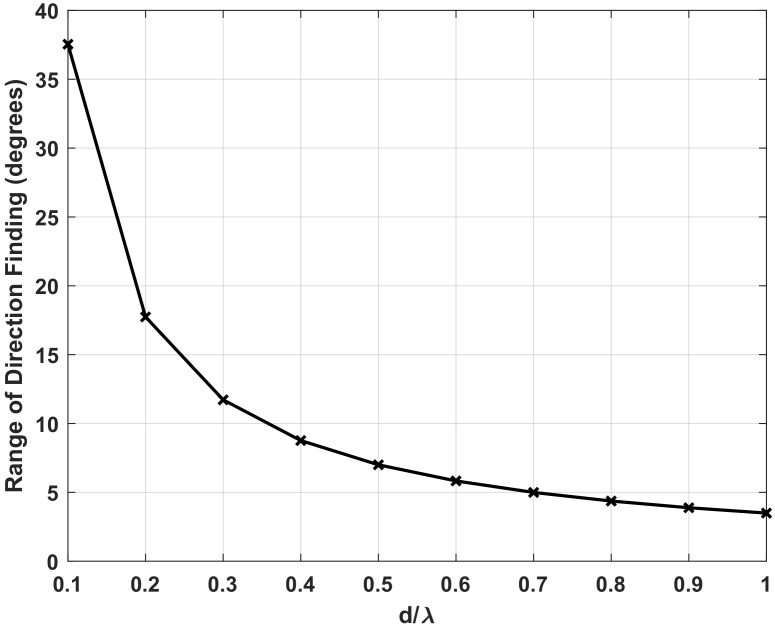
The relationship between the range of direction-finding and the length of baseline.

The CRLB of biologically inspired DOA estimation error for a two-antenna array is derived in [36]. To demonstrate the effect of coupling, we compare the CRLB of the biologically inspired coupled array with the standard antenna array assuming zero coupling, i.e., $H_{I}\left(j\omega_{c}\right)=I_{M}$. We define ϵ as the theoretical accuracy improvement brought by the biologically inspired coupling:

(4) $$\epsilon \left(\theta \right)=\sqrt{\frac{{\mathrm{CRLB}}_{S}\left(\theta \right)}{{\mathrm{CRLB}}_{B}\left(\theta \right)}}$$

where ${\mathrm{CRLB}}_{S}\left(\theta \right)$ and ${\mathrm{CRLB}}_{B}\left(\theta \right)$ are the CRLB of DOA estimation error for standard array and biologically inspired coupled array. When ϵ>1, the theoretical performance of coupled array is better than the standard array, whereas for ϵ<1, the relation will be opposite. We notice that the theoretical accuracy improvement is greater than 1 when the incident angle evaluates in a certain range, which also means the improvement is obtained at the expense of sacrificing the range of direction-finding. Changing the length of the baseline, we can examine the range of direction-finding with different array aperture. Figure 2 demonstrates that the biologically inspired coupled structure is suitable for short baseline scenarios, whereas the range of direction-finding for long baseline decreased significantly. Since the large-aperture array is widely used for multiple-signal resolution and high-accuracy DOA estimation, this phenomenon will greatly restrict the application for biologically inspired coupling.

## 3. Biologically Inspired Fully Coupled Array

**Figure 3:**
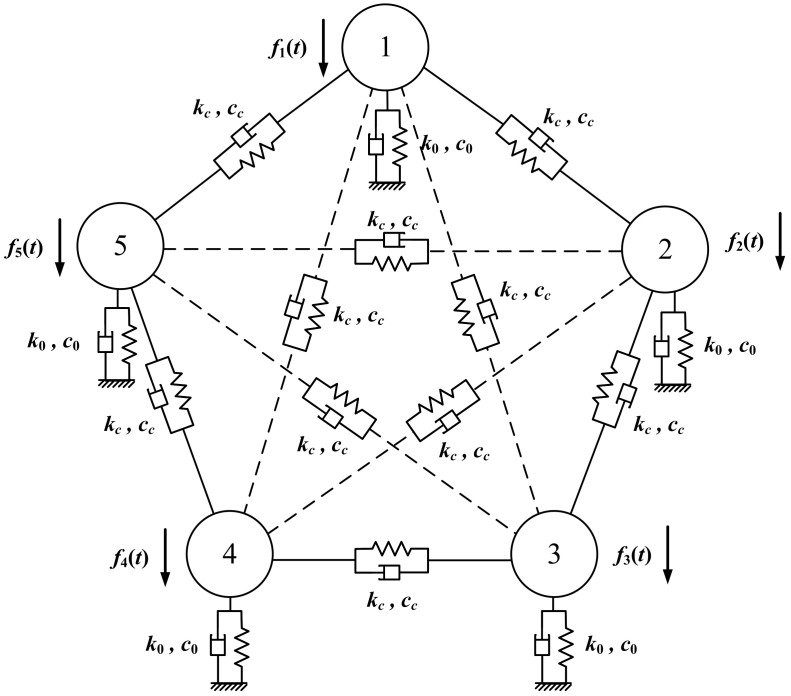
Schematic of five-sensor mechanical coupled structure. The coupling exists between each pair of sensors.

In this section, the coupled structure of the Ormia Ochracea is extended to the *M* sensors array with a similar mechanical principle. In [37], the author proposed a coupled structure such that each sensor is coupled to its immediate neighboring sensors, whereas in this paper, we assume that the coupling exists between each pair of sensors. The mechanical model of five sensors coupled structure is given in Figure 3. The rigid bars connecting each sensor are omitted in this picture. The solid lines correspond to the coupling between adjacent sensors, whereas the dotted lines correspond to the rest of the coupling. The corresponding transfer function of a *M* sensors array can be generalized as a matrix:

(5) $$H_{I}{\left(j\omega_{c}\right)}^{-1}=\left[\begin{array}{cccc} \tilde{D}\left(j\omega_{c}\right) & \tilde{N}\left(j\omega_{c}\right) & \cdots & \tilde{N}\left(j\omega_{c}\right)\\ \tilde{N}\left(j\omega_{c}\right) & \tilde{D}\left(j\omega_{c}\right) & \cdots & \tilde{N}\left(j\omega_{c}\right)\\ \; & \vdots & \vdots & \\ \tilde{N}\left(j\omega_{c}\right) & \tilde{N}\left(j\omega_{c}\right) & \cdots & \tilde{D}\left(j\omega_{c}\right) \end{array}\right]$$

where $\tilde{D}\left(j\omega_{c}\right)$ and $\tilde{N}\left(j\omega_{c}\right)$ can be expressed as:

(6) $$\begin{array}{cc} \tilde{D}\left(j\omega_{c}\right) & =-m_{0}\omega_{c}^{2}+\left[c_{0}+\left(M-1\right)c_{c}\right]j\omega_{c}+k_{0}+\left(M-1\right)k_{c}\\ \tilde{N}\left(j\omega_{c}\right) & =c_{c}j\omega_{c}+k_{c} \end{array}$$

It can be noticed that the coupled structure for multiple sensors is complicated, especially when the number of sensors is increased. Fortunately, as discussed in [36], the coupled structure can be implemented in digital form with less power attenuation under the same phase amplification ability. Thus, we implement the coupled structure for antenna arrays as a multiple-input multiple-output filter. The signals received by each antenna are coupled together, leading to the amplification of the amplitude and phase difference between the output signals.

According to the narrowband assumption mentioned in the final part of Section 1, the incoming signals can be approximated as a summation of components that are pure in frequency, i.e., $s\left(t\right)=\sum_{g=1}^{G_{s}}s^{g}\left(t\right)$, where $G_{s}$ is the number of such pure frequency components. Both s(t) and $s^{g}\left(t\right)$ are Q×1 vectors, which indicates that *Q* signals incident to the array simultaneously.

Since convolution of the biologically inspired coupled filter with the above component results in the multiplication of the time domain incoming signals with the coupled filter computed at the corresponding frequencies of the components [48], the output of the coupled structure for the *g*-th component is then given as

(7) $$y^{g}\left(t\right)=\left[\begin{array}{c} y_{1}^{g}\left(t\right)\\ \vdots \\ y_{M}^{g}\left(t\right) \end{array}\right]=H_{I}\left(j\omega_{g}\right)\left[\begin{array}{c} x_{1}^{g}\left(t\right)\\ \vdots \\ x_{M}^{g}\left(t\right) \end{array}\right]=H_{I}\left(j\omega_{g}\right)\cdot \left[A\left(\theta \right)s^{g}\left(t\right)+e_{e}^{g}\left(t\right)+e_{a}^{g}\left(t\right)\right]$$

where $A\left(\theta \right)=[a\left(\theta_{1}\right)\cdots a\left(\theta_{Q}\right)]$ is the array manifold with θ as the DOA vector for the incoming signals. $\omega_{g}$ is the signal frequency of the *g*-th component. $e_{e}^{g}\left(t\right)$ and $e_{a}^{g}\left(t\right)$ are the M×1 vectors represent the environment noises and amplifier noises corresponding to the *g*-th component. Assuming that the difference of $H_{I}\left(j\omega_{g}\right)$ is ignorable in the range of narrow bandwidth, whose value can be approximated to $H_{I}\left(j\omega_{c}\right)$. Then, the overall output of the coupled structure is given by

(8) $$\begin{array}{cc} y(t) & =\sum_{g=1}^{G_{s}}H_{I}\left(j\omega_{g}\right)\cdot \left[A\left(\theta \right)s^{g}\left(t\right)+e_{e}^{g}\left(t\right)+e_{a}^{g}\left(t\right)\right]\\ \; & \approx H_{I}\left(j\omega_{c}\right)\cdot \sum_{g=1}^{G_{s}}\left[A\left(\theta \right)s^{g}\left(t\right)+e_{e}^{g}\left(t\right)+e_{a}^{g}\left(t\right)\right]\\ \; & =H_{I}\left(j\omega_{c}\right)\cdot \left[A\left(\theta \right)\sum_{g=1}^{G_{s}}s^{g}\left(t\right)+\sum_{g=1}^{G_{s}}e_{e}^{g}\left(t\right)+\sum_{g=1}^{G_{s}}e_{a}^{g}\left(t\right)\right]\\ \; & =H_{I}\left(j\omega_{c}\right)\left[A\left(\theta \right)s\left(t\right)+e_{e}\left(t\right)+e_{a}\left(t\right)\right] \end{array}$$

where $e_{e}\left(t\right)$ and $e_{a}\left(t\right)$ are the summed environment noises and amplifier noises. In the following analysis and simulations, the bandwidth is evaluated to ensure the correctness of (8).

It should be noticed that (8) is free of any array structure constraint; however, we focus on the ULA in this section. The extension to UCA is proposed in Section 6. Hence, the array manifold is given as

(9) $$A\left(\theta \right)=\left[\begin{array}{ccc} 1 & \cdots & 1\\ e^{-j\frac{2\pi d\sin\theta_{1}}{\lambda }} & \cdots & e^{-j\frac{2\pi d\sin\theta_{Q}}{\lambda }}\\ \vdots & \; & \vdots \\ e^{-j\frac{2\pi \left(M-1\right)d\sin\theta_{1}}{\lambda }} & \cdots & e^{-j\frac{2\pi \left(M-1\right)d\sin\theta_{Q}}{\lambda }} \end{array}\right]$$

where *d* is the distance between the adjacent antennas and λ represents the signal wavelength. In this section, the first antenna is set as the reference, thus its corresponding baseline length equals to zero.

It can be seen that the environment and amplifier noises are coupled with the incoming signals together in (8), which brings the correlation to the output noises. Meanwhile, unknown-colored noises may be received from the environment as well. Therefore, an algorithm with unknown covariance matrix of the received noises must be applied in the following sections.

To formulate the DOA estimation problem conveniently, we introduce the statistical assumption corresponding to the model.

- the source vector s(t) follows a zero-mean Gaussian distribution with unknown Q×Q covariance matrix $R_{s}$.
- the spatial colored environment noise $e_{e}\left(t\right)$ is zero-mean Gaussian distributed with unknown covariance matrix $P_{e}$. The matrix is parameterized by the $M^{2}\times 1$ real-valued vector whose elements are denoted as ${\langle P_{e}\rangle }_{mm}$, $R[{\langle P_{e}\rangle }_{mn}]$ and $I[{\langle P_{e}\rangle }_{mn}]$, where ${\langle \cdot \rangle }_{mn}$ is the matrix element in *m*-th row, *n*-th column and m>n. On the other hand, the amplifier noise is considered to be homogeneous white Gaussian noise generally, whose covariance matrix is equal to $P_{a}=\sigma_{a}^{2}I_{M}$, where $\sigma_{a}^{2}$ is the unknown variance of amplifier noise [37]. In addition, the environment noise is assumed to be independent of the amplifier noise.
- s(t), $e_{e}\left(t\right)$ and $e_{a}\left(t\right)$ are uncorrelated in different snapshots.

## 4. Proposed Algorithm

In this section, an iterative FOC-based DOA estimation method with fully coupled array (IFOCE-FC) is proposed to demonstrate the application of biologically inspired coupling. The method consists of two parts: an iterative phase adjustment strategy used to expand the range of biologically inspired direction-finding, and a FOC-based estimation approach used to estimate the DOA with the correlated received noises. The basic idea of this method is changing the orientation of the mainlobe to aim at the incident angle. Therefore, the DOA to be estimated would fall within the range of biologically inspired direction-finding and the estimation accuracy can be improved by biologically inspired coupling. It is an iterative process that the DOA can be estimated with better accuracy in each iteration.

### 4.1. Algorithm Description

Since the IFOCE-FC is an iterative method, we assume that the *m*-th iteration is proceeded currently. We define the incident angle of *j*-th signal in the *m*-th iteration as $\theta_{j}^{\left(m\right)}$ and the input signals of biologically inspired coupled structure as $x^{\left(m\right)}\left(t\right)$. Hence, the corresponding output signals can be obtained by ${\tilde{y}}^{\left(m\right)}\left(t\right)=H_{I}\left(j\omega_{c}\right)x^{\left(m\right)}\left(t\right)$. The incident angle $\theta_{j}^{\left(m\right)}$ can be estimated by the FOC of ${\tilde{y}}^{\left(m\right)}\left(t\right)$ when the correlated noise exists, whose covariance matrix is unknown.

The FOC matrix can be estimated by the finite samples of ${\tilde{y}}^{\left(m\right)}\left(t\right)$, which is given by

(10) $$\begin{array}{cc} {\hat{C}}^{\left(m\right)}= & \frac{1}{N}\sum_{n=1}^{N}\left({\tilde{y}}^{\left(m\right)}\left(t\right)⊗{\left({\tilde{y}}^{\left(m\right)}\left(t\right)\right)}^{*}\right){\left({\tilde{y}}^{\left(m\right)}\left(t\right)⊗{\left({\tilde{y}}^{\left(m\right)}\left(t\right)\right)}^{*}\right)}^{H}\\ \; & -\left[\frac{1}{N}\sum_{n=1}^{N}{\tilde{y}}^{\left(m\right)}\left(t\right)⊗{\left({\tilde{y}}^{\left(m\right)}\left(t\right)\right)}^{*}\right]\cdot \left[\frac{1}{N}\sum_{n=1}^{N}{\left({\tilde{y}}^{\left(m\right)}\left(t\right)⊗{\left({\tilde{y}}^{\left(m\right)}\left(t\right)\right)}^{*}\right)}^{H}\right]\\ \; & -\left[\frac{1}{N}\sum_{n=1}^{N}{\tilde{y}}^{\left(m\right)}\left(t\right){\left({\tilde{y}}^{\left(m\right)}\left(t\right)\right)}^{H}\right]⊗\left[\frac{1}{N}\sum_{n=1}^{N}{\tilde{y}}^{\left(m\right)}\left(t\right){\left({\tilde{y}}^{\left(m\right)}\left(t\right)\right)}^{H}\right] \end{array}$$

where *N* is the number of samples.

It can be noticed that ${\hat{C}}^{\left(m\right)}$ is a $Q^{2}\times Q^{2}$ matrix, whose rank is equal to $Q^{2}$. On all accounts, ${\hat{C}}^{\left(m\right)}$ is a Hermitian matrix, but not positive definite. The eigenvalues of ${\hat{C}}^{\left(m\right)}$ can be divided into two groups, in which $M^{2}-Q^{2}$ of them are related to the received noises, whereas the rest relate to incoming signals with arbitrary signs.

The signal and noise subspace can be obtained by the singular value decomposition (SVD) of the FOC matrix [40,42], which can be expressed as

(11) $${\hat{C}}^{\left(m\right)}=\left[\begin{array}{cc} U_{S} & U_{E} \end{array}\right]\left[\begin{array}{cc} \Sigma_{S} & \\ \; & \Sigma_{E} \end{array}\right]\left[\begin{array}{c} V_{S}\\ V_{E} \end{array}\right]$$

where $\Sigma_{S}$ and $\Sigma_{E}$ denote the diagonal matrix containing the singular values of ${\hat{C}}^{\left(m\right)}$. $U_{S}$ and $U_{E}$ are the matrices given the left singular vectors as their columns, whereas $V_{S}$ and $V_{E}$ given the right singular vectors. It is known that the columns of $U_{E}$ are orthogonal to the columns of $\left[H_{I}\left(j\omega_{c}\right)A\left(\theta \right)\right]⊗{\left[H_{I}\left(j\omega_{c}\right)A\left(\theta \right)\right]}^{*}$. Using this property, the DOA can be estimated by the minimization of the null spectrum:

(12) $${\hat{\theta }}_{j}^{\left(m\right)}=\underset{\theta }{\arg\min}F\left(\theta \right)=\underset{\theta }{\arg\min}{\left[\bar{a}\left(\theta \right)⊗\bar{a}{\left(\theta \right)}^{*}\right]}^{H}U_{E}U_{E}^{H}\left[\bar{a}\left(\theta \right)⊗\bar{a}{\left(\theta \right)}^{*}\right]$$

where $\bar{a}\left(\theta \right)=H_{I}\left(j\omega_{c}\right)a\left(\theta \right)$. Equivalently, the DOA can be estimated by the maximization of 1/F(θ) as well.

To reduce the incident angle in the next iteration, a phase adjustment strategy is applied to $x^{\left(m\right)}\left(t\right)$. Using the estimated result ${\hat{\theta }}_{j}^{\left(m\right)}$, the corresponding phase difference between the antenna and reference is

(13) $${\hat{\phi }}_{i,j}^{\left(m\right)}=\frac{2\pi \left(i-1\right)d\sin{\hat{\theta }}_{j}^{\left(m\right)}}{\lambda }$$

where the subscript *i* denotes the *i*-th antenna.

Then, we define a phase adjustment matrix $\Psi_{j}^{\left(m\right)}$, which is given by

(14) $$\Psi_{j}^{\left(m\right)}=\mathrm{diag}\left(\Psi_{j}^{\left(m\right)}\right)=\left[\begin{array}{cccc} e^{j{\hat{\phi }}_{1,j}^{\left(m\right)}} & \; & & \\ \; & e^{j{\hat{\phi }}_{2,j}^{\left(m\right)}} & \; & \\ \; & \; & \ddots & \\ \; & \; & & e^{j{\hat{\phi }}_{M,j}^{\left(m\right)}} \end{array}\right]$$

where the diagonal of $\Psi_{j}^{\left(m\right)}$ is obtained by the estimation of ${\hat{\phi }}_{i,j}^{\left(m\right)}$, which also means $\Psi_{j}^{\left(m\right)}={\left[e^{j{\hat{\phi }}_{1,j}^{\left(m\right)}},e^{j{\hat{\phi }}_{2,j}^{\left(m\right)}},\cdots ,e^{j{\hat{\phi }}_{M,j}^{\left(m\right)}}\right]}^{T}$.

Hence, the input and output signals of biologically inspired coupled structure for (m+1)-th iteration are

(15) $$\begin{array}{cc} x^{\left(m+1\right)}\left(t\right) & \;\;\;\;\;=\Psi_{j}^{\left(m\right)}x^{\left(m\right)}\left(t\right) \end{array}$$

(16) $$\begin{array}{cc} {\tilde{y}}^{\left(m+1\right)}\left(t\right) & =H_{I}\left(j\omega_{c}\right)x^{\left(m+1\right)}\left(t\right)=H_{I}\left(j\omega_{c}\right)\Psi_{j}^{\left(m\right)}x^{\left(m\right)}\left(t\right) \end{array}$$

According to (9) and (15), we can obtain a more detailed expression

(17) $$\begin{array}{cc} x^{\left(m+1\right)}\left(t\right) & =\Psi_{j}^{\left(m\right)}x^{\left(m\right)}\left(t\right)=\Psi_{j}^{\left(m\right)}A\left(\theta^{\left(m\right)}\right)s\left(t\right)\\ \; & =\left[\begin{array}{cccc} 1 & \; & & \\ \; & e^{j\frac{2\pi d\sin{\hat{\theta }}_{j}^{\left(m\right)}}{\lambda }} & \; & \\ \; & \; & \ddots & \\ \; & \; & & e^{j\frac{2\pi \left(M-1\right)d\sin{\hat{\theta }}_{j}^{\left(m\right)}}{\lambda }} \end{array}\right]\left[\begin{array}{ccc} \cdots & 1 & \cdots \\ \; & e^{-j\frac{2\pi d\sin\theta_{j}^{\left(m\right)}}{\lambda }} & \\ \; & \vdots & \\ \cdots & e^{-j\frac{2\pi \left(M-1\right)d\sin\theta_{j}^{\left(m\right)}}{\lambda }} & \cdots \end{array}\right]s\left(t\right)\\ \; & =\left[\begin{array}{ccc} \cdots & 1 & \cdots \\ \; & e^{-j\frac{2\pi d\left(\sin\theta_{j}^{\left(m\right)}-\sin{\hat{\theta }}_{j}^{\left(m\right)}\right)}{\lambda }} & \\ \; & \vdots & \\ \cdots & e^{-j\frac{2\pi \left(M-1\right)d\left(\sin\theta_{j}^{\left(m\right)}-\sin{\hat{\theta }}_{j}^{\left(m\right)}\right)}{\lambda }} & \cdots \end{array}\right]s\left(t\right)=A\left(\theta^{\left(m+1\right)}\right)s\left(t\right) \end{array}$$

Therefore, the corresponding incident angle of *j*-th signal for $x^{\left(m+1\right)}\left(t\right)$ is given by

(18) $$\sin\theta_{j}^{\left(m+1\right)}=\sin\theta_{j}^{\left(m\right)}-\sin{\hat{\theta }}_{j}^{\left(m\right)}$$

It can be seen from (18) that the phase adjustment strategy is essentially changing the orientation of the mainlobe and reducing the included angle between the mainlobe and original incident angle. In this case, it is more likely to locate in the range of biologically inspired direction-finding.

We assume that the IFOCE-FC method terminates at *k*-th iteration according to the termination condition introduced in the next part. Thus, the total phase adjustment matrix $\Psi_{j}$ can be expressed as

(19) $$\Psi_{j}=\prod_{m=0}^{k-1}\Psi_{j}^{\left(m\right)}$$

The original incident angle $\theta_{j}$ can be estimated by the iteration results mentioned above:

(20) $${\hat{\theta }}_{j}=\arcsin\left(\sum_{m=0}^{k}\sin{\hat{\theta }}_{j}^{\left(m\right)}\right)$$

The initial input signals $x^{\left(0\right)}\left(t\right)$ are equal to the original array outputs x(t) without phase adjustment, whereas $\theta_{j}^{\left(0\right)}$ represents the original incident angle $\theta_{j}$. However, it should be pointed out here that $\theta_{j}$ may not locate the range of biologically inspired direction-finding, hence the DOA estimation method based on standard array has better performance than the biologically inspired coupled array in the first iteration step. We can obtain ${\hat{\theta }}_{j}^{\left(0\right)}$ by (12) with $H_{I}\left(j\omega_{c}\right)$ set as an identity matrix $I_{M}$.

**Figure 4:**
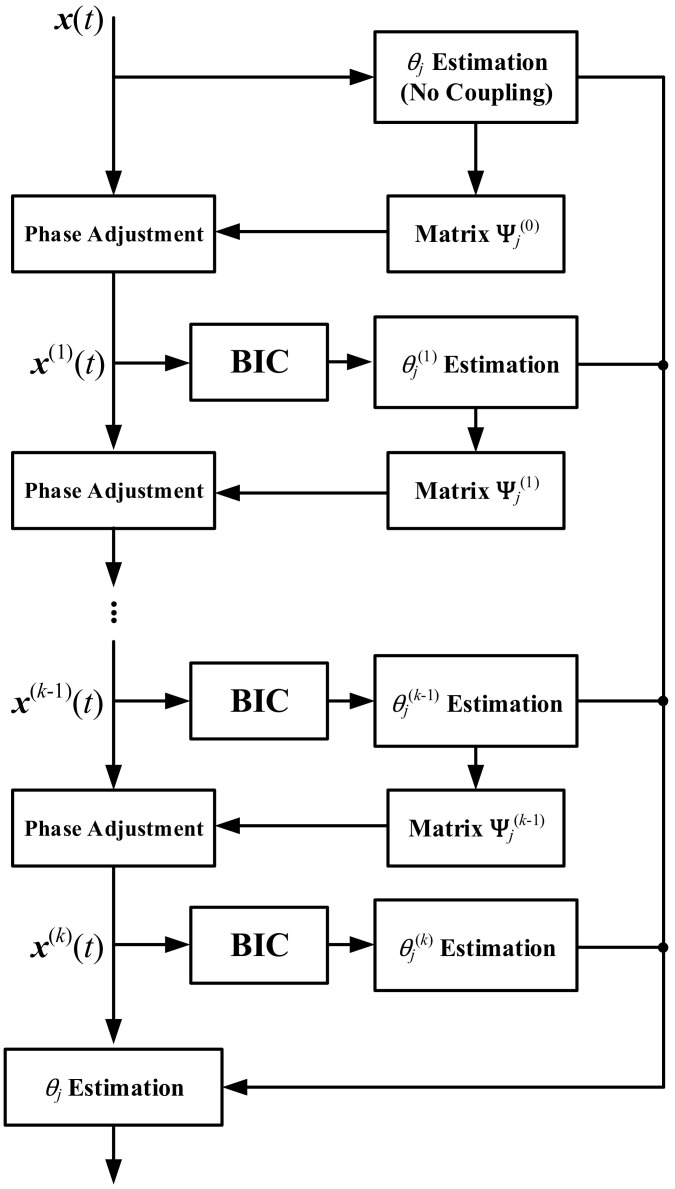
Flow chart corresponding to the IFOCE-FC method, where BIC denotes the module of biologically inspired coupling.

Please note that the phase adjustment matrices are fixed in the following iterations once they have been obtained; thus, the estimation error of $\theta_{j}$ is merely determined by the estimation result in the final iteration, which means the accuracy does improve by biologically inspired coupling. Figure 4 demonstrates the flow chart of the IFOCE-FC method.

For multiple incoming signals, a different phase adjustment matrix $\Psi_{j}$, j=1⋯Q can be obtained. Then, the DOA is estimated by (20) under the corresponding phase adjustment, respectively.

### 4.2. Convergence Analysis

As for an iterative algorithm, convergence analysis needs to be executed. As we analyzed earlier, the iteration process is repeated with the decreased incident angle $\theta_{j}^{\left(m\right)}$. Hence, in this part, we analyze the probability density function (PDF) of the incident angle in each iteration and give its termination condition.

Let

(21) $$\begin{array}{cc} \zeta_{j}^{\left(m\right)} & =\sin\theta_{j}^{\left(m\right)}\\ {\hat{\zeta }}_{j}^{\left(m\right)} & =\sin{\hat{\theta }}_{j}^{\left(m\right)} \end{array}$$

We assume that ${\hat{\zeta }}_{j}^{\left(0\right)}$ follows the Gaussian distribution, with the mean equal to $\sin\theta_{j}$ and the standard deviation is denoted as $\sigma_{j}$. Then, according to (18), $\zeta_{j}^{\left(1\right)}$ for $x^{\left(1\right)}\left(t\right)$ is zero-mean Gaussian distributed with standard deviation equal to $\sigma_{j}$.

(22) $$f_{1}\left(\zeta_{j}^{\left(1\right)}\right)=\frac{1}{\sigma_{j}\sqrt{2\pi }}\exp\left(-\frac{\zeta_{j}^{{\left(1\right)}^{2}}}{2\sigma_{j}^{2}}\right)$$

Estimation of $\zeta_{j}^{\left(1\right)}$ is obtained by biologically inspired direction-finding as an unbiased estimator and its standard deviation equals $\sigma_{j}/\epsilon \left(\arcsin\zeta_{j}^{\left(1\right)}\right)$.

In the *m*-th (m≥2) iteration step, the prior PDF of $\zeta_{j}^{\left(m-1\right)}$ is defined as $f_{m-1}\left(\zeta_{j}^{\left(m-1\right)}\right)$. Similarly, the conditional PDF for ${\hat{\zeta }}_{j}^{\left(m-1\right)}$ under specific $\zeta_{j}^{\left(m-1\right)}$ is

(23) $$f_{{\mathrm{est}}_{m-1}}\left({\hat{\zeta }}_{j}^{\left(m-1\right)}|\zeta_{j}^{\left(m-1\right)}\right)=\frac{\epsilon (\arcsin\zeta_{j}^{\left(m-1\right)})}{\sigma_{j}\sqrt{2\pi }}e^{-\frac{{\left({\hat{\zeta }}_{j}^{\left(m-1\right)}-\zeta_{j}^{\left(m-1\right)}\right)}^{2}\epsilon {\left(\arcsin\zeta_{j}^{\left(m-1\right)}\right)}^{2}}{2\sigma_{j}^{2}}}$$

where its standard deviation is decreased by the biologically inspired coupling with the scaling factor $\epsilon (\arcsin\zeta_{j}^{\left(m-1\right)})$.

Then we can evaluate $\zeta_{j}^{\left(m\right)}$ and its conditional PDF is given by

(24) $$f_{m|m-1}\left(\zeta_{j}^{\left(m\right)}|\zeta_{j}^{\left(m-1\right)}\right)=\frac{\epsilon (\arcsin\zeta_{j}^{\left(m-1\right)})}{\sigma_{j}\sqrt{2\pi }}\exp\left[-\frac{{\left(\zeta_{j}^{\left(m\right)}\epsilon \left(\arcsin\zeta_{j}^{\left(m-1\right)}\right)\right)}^{2}}{2\sigma_{j}^{2}}\right]$$

Considering all the possible values for $\zeta_{j}^{\left(m-1\right)}$, the prior PDF of $\zeta_{j}^{\left(m\right)}$ can be expressed as:

(25) $$f_{m}\left(\zeta_{j}^{\left(m\right)}\right)=\int_{-1}^{1}f_{m|m-1}\left(\zeta_{j}^{\left(m\right)}|\zeta_{j}^{\left(m-1\right)}\right)f_{m-1}\left(\zeta_{j}^{\left(m-1\right)}\right)d\zeta_{j}^{\left(m-1\right)}$$

**Figure 5:**
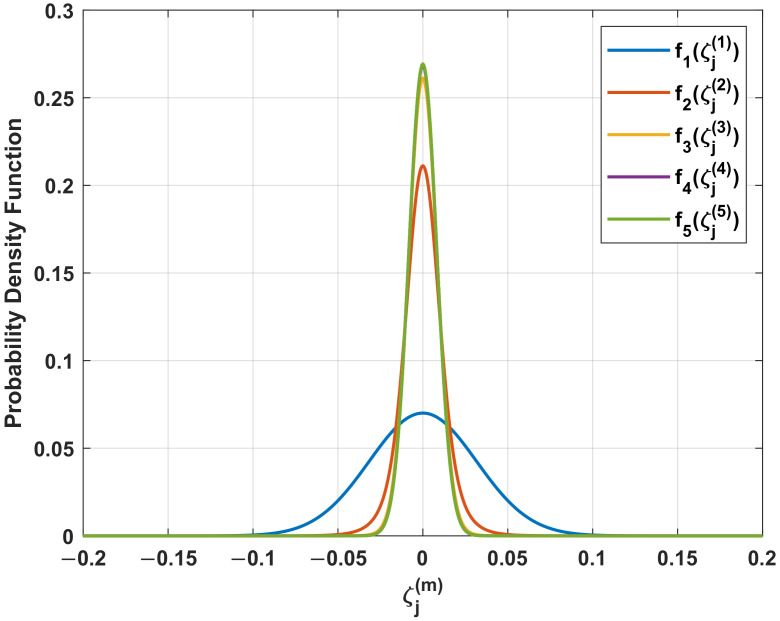
The probability density function of $\zeta_{j}^{\left(m\right)}$ in each iteration.

Please note that the probability distribution shown in (25) changes in each iteration. Hence, we can analyze the convergence of iteration in the perspective of probability. It can be terminated when the change of PDF between adjacent iterations is ignorable. The prior PDFs of $\zeta_{j}^{\left(m\right)}$ in each iteration are plotted in Figure 5 with the SNR of received signals equal to 5 dB and $d=\frac{\lambda }{2}$. We can observe that the prior PDF becomes sharper as the standard deviation decreases significantly when the iteration step is less than 5. The prior PDF remains unchanged when the number of iterations is greater than 5. Although the estimation result may be different if the iteration keeps going, the accuracy remains unchanged. In this manner, the iteration can be considered to be converged. It should be pointed out here that the convergence is analyzed in terms of estimation performance, rather than the estimation results.

**Figure 6:**
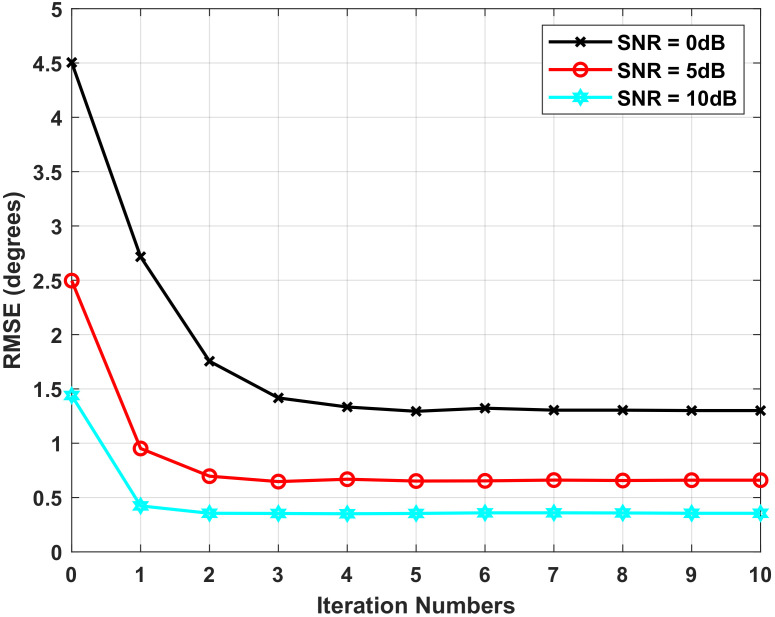
The RMSE of DOA estimation versus the number of iterations.

We further examine the estimation performance of the proposed algorithm with different numbers of iterations. The estimation error is measured by the root mean-square error (RMSE), which is defined as

(26) $$\mathrm{RMSE}\left(\theta_{j}\right)=\sqrt{\frac{1}{M_{c}}\sum_{n=1}^{M_{c}}{\left({\hat{\theta }}_{j,n}-\theta_{j}\right)}^{2}}$$

where ${\hat{\theta }}_{j,n}$ is the DOA estimation for *j*-th signal in the *n*-th simulation. $M_{c}$ denotes the number of Monte Carlo simulations and we take $M_{c}=500$. Let $\theta =30^{\circ }$ and the iteration number vary from 0 to 10. Figure 6 shows the resulting RMSE with various SNR. It can be seen that the algorithm converges quickly under the given conditions. Furthermore, the algorithm converges faster when the SNR increases.

In general, the iteration terminates when at least one of the following conditions are met:

- the variation of prior PDF between the adjacent iterations drops below a threshold $\xi_{\mathrm{th}1}$: $\int_{-1}^{1}{\left|f_{m}\left(\zeta \right)-f_{m-1}\left(\zeta \right)\right|}^{2}d\zeta \leq \xi_{\mathrm{th}1}$
- the number of iterations reaches the maximum threshold $m_{\max}$: $m\geq m_{\max}$

## 5. CRLB

Inspired by [49], we present the derivation of CRLB for the DOA estimation error considering the phase adjustment to the received signals. First, we define the output signals of the coupled structure when the phase adjustment terminates as $\tilde{y}\left(t\right)$ and we can obtain its covariance matrix, which is determined as

(27) $$R=E\left[\tilde{y}\left(t\right)\tilde{y}{\left(t\right)}^{H}\right]=\tilde{A}\left(\theta \right)R_{s}\tilde{A}{\left(\theta \right)}^{H}+\Gamma$$

where $\tilde{A}\left(\theta \right)$ and Γ equal to

(28) $$\tilde{A}\left(\theta \right)=H_{I}\left(j\omega_{c}\right)\Psi_{j}A\left(\theta \right)={\tilde{H}}_{I}\left(j\omega_{c}\right)A\left(\theta \right)$$

(29) $$\Gamma =H_{I}\left(j\omega_{c}\right)\Psi_{j}\left(P_{e}+\sigma_{a}^{2}I\right)\Psi_{j}^{H}H_{I}{\left(j\omega_{c}\right)}^{H}={\tilde{H}}_{I}\left(j\omega_{c}\right)\left(P_{e}+\sigma_{a}^{2}I\right){\tilde{H}}_{I}{\left(j\omega_{c}\right)}^{H}$$

with ${\tilde{H}}_{I}\left(j\omega_{c}\right)=H_{I}\left(j\omega_{c}\right)\Psi_{j}$. Since the environment noise is assumed to be spatially nonuniform, the coupled noise can be statistically correlated accordingly. Hence, both $P_{e}$ and Γ are non-diagonal matrix.

Then, we denote the unknown parameters as η

(30) $$\eta ={\left[\theta^{T}\;r_{s}^{T}\;\gamma^{T}\right]}^{T}$$

where $r_{s}$ contains the real and imaginary parts of the elements in $R_{s}$ and γ corresponds to the elements of Γ.

According to the conclusion in [50], the Fisher information matrix of η for stochastic incoming signals is given by

(31) $$J\left(\eta \right)=N\cdot {\left(\frac{\partial r_{v}}{\partial \eta^{T}}\right)}^{H}\cdot \left[{\left(R^{-1}\right)}^{T}⊗R^{-1}\right]\cdot \frac{\partial r_{v}}{\partial \eta^{T}}$$

where $r_{v}$ is the vectorization of R. This operation is denoted as vec(·) and $r_{v}$ can be expressed as

(32) $$r_{v}=\mathrm{vec}\left(R\right)=\sum_{n=1}^{M}B_{n}Re_{n}$$

where $B_{n}$ is a $M^{2}\times M$ column-wise block matrix, with an identity matrix only in the *n*-th block and others are zeros. $e_{n}$ is the *n*-th canonical basis vector, with *n*-th element equal to 1 and others are 0.

Using (27) and (29), we can calculate $r_{v}$ as:

(33) $$\begin{array}{cc} r_{v}= & \mathrm{vec}\left(R\right)=\mathrm{vec}\left(\tilde{A}\left(\theta \right)R_{s}\tilde{A}{\left(\theta \right)}^{H}+\Gamma \right)=\left[\tilde{A}{\left(\theta \right)}^{*}⊗\tilde{A}\left(\theta \right)\right]\cdot \mathrm{vec}\left(R_{s}\right)+\mathrm{vec}\left(\Gamma \right)\\ = & \left[\tilde{A}{\left(\theta \right)}^{*}⊗\tilde{A}\left(\theta \right)\right]\cdot \mathrm{vec}\left(R_{s}\right)+\left[{\tilde{H}}_{I}{\left(j\omega_{c}\right)}^{*}⊗{\tilde{H}}_{I}\left(j\omega_{c}\right)\right]\cdot [\mathrm{vec}\left(P_{e}\right)+\sigma_{a}^{2}\cdot \mathrm{vec}\left(I_{M}\right)] \end{array}$$

We can obtain $\left[{\left(R^{-\frac{1}{2}}\right)}^{T}⊗R^{-\frac{1}{2}}\right]\cdot \frac{\partial r_{v}}{\partial \eta^{T}}$, which is written as a row-wise block matrix

(34) $$\left[{\left(R^{-\frac{1}{2}}\right)}^{T}⊗R^{-\frac{1}{2}}\right]\cdot \frac{\partial r_{v}}{\partial \eta^{T}}=\left[{\left(R^{-\frac{1}{2}}\right)}^{T}⊗R^{-\frac{1}{2}}\right]\cdot \left[\frac{\partial r_{v}}{\partial \theta^{T}}\;|\;\frac{\partial r_{v}}{\partial r_{s}^{T}}\;\frac{\partial r_{v}}{\partial \gamma^{T}}\right]=[G\;|\;W]$$

where the partial derivatives are evaluated as follows

(35) $$\frac{\partial r_{v}}{\partial \theta^{T}}=\left[\frac{\partial \tilde{A}{\left(\theta \right)}^{*}}{\partial \theta^{T}}⊗\tilde{A}\left(\theta \right)+\tilde{A}{\left(\theta \right)}^{*}⊗\frac{\partial \tilde{A}\left(\theta \right)}{\partial \theta^{T}}\right]\cdot \mathrm{vec}\left(R_{s}\right)$$

(36) $$\frac{\partial r_{v}}{\partial r_{s}^{T}}=\left[\tilde{A}{\left(\theta \right)}^{*}⊗\tilde{A}\left(\theta \right)\right]\cdot \frac{\partial \mathrm{vec}(R_{s})}{\partial r_{s}^{T}}=\left[\tilde{A}{\left(\theta \right)}^{*}⊗\tilde{A}\left(\theta \right)\right]\cdot X$$

(37) $$\frac{\partial r_{v}}{\partial \gamma^{T}}=\frac{\partial \mathrm{vec}(\Gamma )}{\partial \gamma^{T}}=Y$$

with the matrix X and Y satisfy the expressions: $\mathrm{vec}\left(R_{s}\right)=Xr_{s}$ and vec(Γ)=Yγ.

Then, the Fisher information matrix in (31) can be rewritten as

(38) $$J\left(\eta \right)=N\cdot \left[\begin{array}{cc} G^{H}G & G^{H}W\\ W^{H}G & W^{H}W \end{array}\right]$$

Using the matrix inversion equation in [51], the CRLB of DOA estimation error is given as

(39) $$\mathrm{CRLB}\left(\theta \right)=\frac{{\left[G^{H}G-G^{H}W{\left(W^{H}W\right)}^{-1}W^{H}G\right]}^{-1}}{N}=\frac{{\left(G^{H}\cdot \Pi^{⊥}\left[W\right]\cdot G\right)}^{-1}}{N}$$

## 6. Algorithms Extension to UCA

In this section, we extend our analysis to the UCA and derive the corresponding CRLB of both azimuth and elevation estimation error.

Similar to the array manifold of ULA in (9), the corresponding matrix for UCA is given as

(40) $$A\left(\varphi ,\beta \right)=\left[\begin{array}{ccc} e^{-j\phi_{1,1}} & \cdots & e^{-j\phi_{1,Q}}\\ e^{-j\phi_{2,1}} & \cdots & e^{-j\phi_{2,Q}}\\ \vdots & \; & \vdots \\ e^{-j\phi_{M,1}} & \cdots & e^{-j\phi_{M,Q}} \end{array}\right]$$

where φ and β are the azimuth and elevation vector for incoming signals. $\phi_{i,j}$ is modified as the phase difference between the *i*-th antenna and reference on the center of the array for *j*-th signal,

(41) $$\phi_{i,j}=\frac{2\pi r\cos\beta_{j}\cos\left(\varphi_{j}-\mu_{i}\right)}{\lambda }$$

where *r* is the radius of UCA and $\mu_{i}$ is the azimuth angle of the *i*-th antenna.

The procedures of estimation are the same as we proposed in Section 4, but a two-dimensional search is required to determine the azimuth and elevation for UCA.

To compute the CRLB of the estimation error for azimuth and elevation, we modify the unknown parameters as

(42) $$\eta ={\left[\varphi^{T}\;\beta^{T}\;r_{s}^{T}\;\gamma^{T}\right]}^{T}$$

Then, the row-wise block matrix in (34) can be modified in the form as

(43) $$\left[{\left(R^{-\frac{1}{2}}\right)}^{T}⊗R^{-\frac{1}{2}}\right]\cdot \frac{\partial r_{v}}{\partial \eta^{T}}=\left[{\left(R^{-\frac{1}{2}}\right)}^{T}⊗R^{-\frac{1}{2}}\right]\cdot \left[\frac{\partial r_{v}}{\partial \varphi^{T}}\;\frac{\partial r_{v}}{\partial \beta^{T}}\;|\;\frac{\partial r_{v}}{\partial r_{s}^{T}}\;\frac{\partial r_{v}}{\partial \gamma^{T}}\right]=[G\;|\;W]$$

The partial derivatives of $r_{v}$ with respect to φ and β are given by

(44) $$\frac{\partial r_{v}}{\partial \varphi^{T}}=\left[\frac{\partial \tilde{A}{\left(\varphi ,\beta \right)}^{*}}{\partial \varphi^{T}}⊗\tilde{A}\left(\varphi ,\beta \right)+\tilde{A}{\left(\varphi ,\beta \right)}^{*}⊗\frac{\partial \tilde{A}\left(\varphi ,\beta \right)}{\partial \varphi^{T}}\right]\cdot \mathrm{vec}\left(R_{s}\right)$$

(45) $$\frac{\partial r_{v}}{\partial \beta^{T}}=\left[\frac{\partial \tilde{A}{\left(\varphi ,\beta \right)}^{*}}{\partial \beta^{T}}⊗\tilde{A}\left(\varphi ,\beta \right)+\tilde{A}{\left(\varphi ,\beta \right)}^{*}⊗\frac{\partial \tilde{A}\left(\varphi ,\beta \right)}{\partial \beta^{T}}\right]\cdot \mathrm{vec}\left(R_{s}\right)$$

Similar to (39), we can obtain the CRLB of both azimuth and elevation estimation error. The diagonal of this matrix contains the minimum possible variance that the azimuth and elevation estimator can achieve.

## 7. Numerical Results

In this section, we present the Monte Carlo simulation results with the existence of multiple signals and spatially colored noise to demonstrate the performance of the proposed algorithm. In this simulation, both the ULA and UCA are used to estimate the DOA. The number of elements for antenna array equals 9, i.e., M=9. The frequency of incoming signals equals 30 MHz, whereas the bandwidth equals 0.3 MHz. Thus, the incoming signals satisfy the narrowband array assumption. The covariance matrix $P_{e}$ of the environment noise is given by

(46) $${\langle P_{e}\rangle }_{mn}=\sigma_{e}^{2}\exp\left[-{\left(m-n\right)}^{2}\xi \right]$$

where $\sigma_{e}^{2}$ is the main diagonal element of $P_{e}$ and ξ=0.3. The value of $\sigma_{e}^{2}$ is determined by the SNR. In this paper, we define the SNR as

(47) $$\mathrm{SNR}=\frac{\mathrm{tr}[A\left(\theta \right)R_{s}A{\left(\theta \right)}^{H}]}{M\sigma_{a}^{2}+\mathrm{tr}\left[P_{e}\right]}$$

It can be observed that Equation (47) reflects the ratio between the average power of received signals and noises. Moreover, we assume that $\sigma_{a}^{2}=\sigma_{e}^{2}$ in these simulations. Therefore, the environment noise and amplifier noise can be generated by the above-mentioned conditions. It should be noticed that the different relationships between noise powers can also be used in these simulations and the proposed algorithm would not be affected by this change.

For the coupled structure, our approach to obtain its parameters is similar to the optimization proposed in [37]. The optimization maximizes the direction-finding performance within the given range of frequency. The main difference between them is the coupling configuration (coupling with neighboring antennas versus coupling between each pair of antennas).

We keep the number of time samples *N* to 128 and the number of Monte Carlo simulations $M_{c}$ to 500. The performance of the proposed algorithm is evaluated in both resolution capability and estimation accuracy. Finally, the computational complexity is compared to the existing algorithms as well.

### 7.1. Resolution Capability

**Figure 7:**
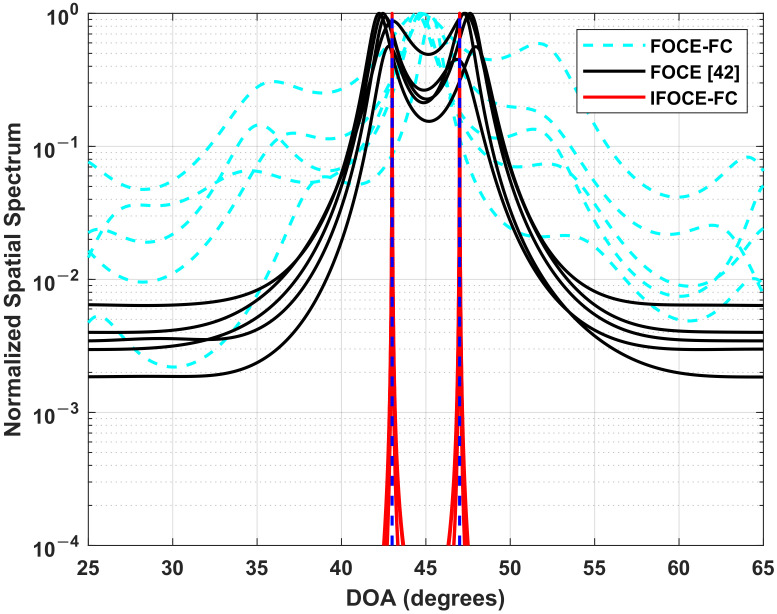
The normalized spatial spectrum for different DOA estimation algorithms in the ULA application.

In this part, we examine the resolution capability of the proposed IFOCE-FC algorithm. For ULA, the distance between neighboring antennas is $\frac{\lambda }{2}$. Two independent signals incident at $43^{\circ }$ and $47^{\circ }$ are received by the array with SNR equal to 5 dB. We conduct 5 independent experiments and plot the reciprocal of the null spectrum F(θ), respectively. We compare the IFOCE-FC algorithm with the FOC-based MUSIC in [42], which is referred to as FOCE and the proposed algorithm without iterative scheme (FOCE-FC). The corresponding normalized spatial spectrum are demonstrated in Figure 7. It can be noticed that the FOCE-FC algorithm cannot distinguish the signals, due to the incident angles outside the range of biologically inspired direction-finding. This leads to the degradation of resolution performance compared to the no coupled array, i.e., FOCE. However, with the effect of the iterative scheme, the IFOCE-FC algorithm has sharper peaks at the incident angles, and it can resolve the signals exactly.

Then, throughout the simulation, we analyze the resolvable angle for different algorithms. First, the resolution criterion was defined in [52] as the following inequality

(48) $$\kappa \left(\theta_{1},\theta_{2}\right)=F\left(\frac{\theta_{1}+\theta_{2}}{2}\right)-\frac{F\left(\theta_{1}\right)+F\left(\theta_{2}\right)}{2}>0$$

**Figure 8:**
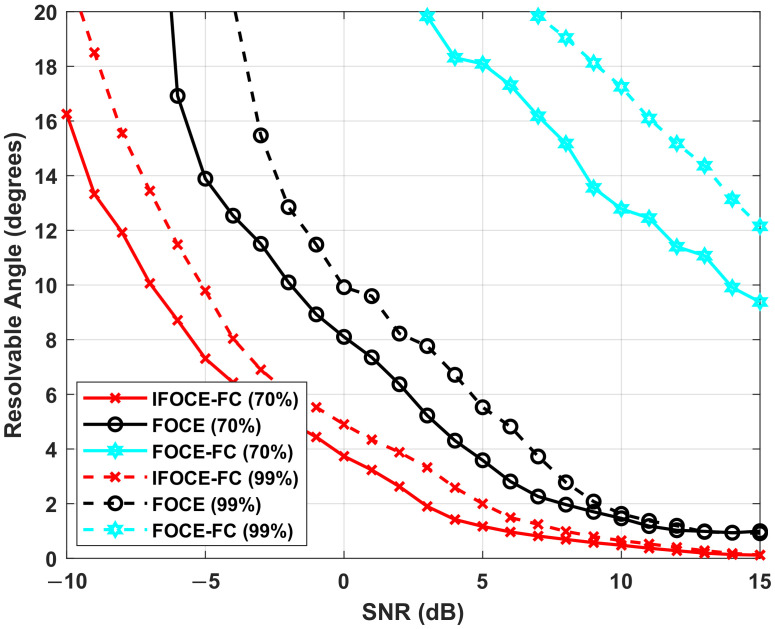
Comparison of different algorithms for the resolvable angle versus the SNR in the ULA application.

The inequality expresses that the null spectrum magnitude at the mid-angle lies above the line segment connecting the valleys corresponding to the two signals. Thus, the signals are considered to be resolvable if the above inequality holds, and irresolvable otherwise. We can count the number of resolutions and obtain the corresponding probability:

(49) $$P_{\mathrm{res}}=\frac{N_{r}}{M_{c}}$$

where $N_{r}$ is the number of simulations that satisfy the inequality $\kappa (\theta_{1},\theta_{2})>0$. We can notice that the resolvable angle is related to the probability of resolution. The practical significance of the resolvable angle here can be described as the minimum angle separation required for a prespecified resolution probability. Thus, the resolvable angle becomes larger when the prespecified resolution probability increased. In this simulation, the resolution probability is specified as 0.7 and 0.99. Suppose that the signals incident to the array from the directions of $45^{\circ }-\frac{\theta_{\mathrm{sep}}}{2}$ and $45^{\circ }+\frac{\theta_{\mathrm{sep}}}{2}$, respectively, where $\theta_{\mathrm{sep}}$ is the angle separation of the incoming signals. We can obtain the resolvable angle with different resolution probability as a function of SNR, which is shown in Figure 8. Obviously, it can be seen that the FOCE-FC algorithm cannot offer satisfactory resolution performance compared with the IFOCE-FC and FOCE algorithms. As analyzed before, the IFOCE-FC algorithm has the best resolution performance; thus, the resolvable angle of this method is the smallest among all the presented algorithms. As SNR increases, the resolvable angle decreased to zero asymptotically. It can be interpreted that the proposed algorithm is super-resolution, thus the signals can be resolved without the existence of noises, whatever the angle separation taken. By contrast, as SNR decreases, the angle separation required increases. A careful examination also shows that the curves become parallel to the vertical axis asymptotically when SNR decreased. These results indicate that the SNR thresholds exist for different algorithms, respectively. The signals cannot be resolved regardless of the value of angle separation when the SNR is less than its threshold. The IFOCE-FC algorithm has the minimum SNR threshold among the presented algorithms in this part.

**Figure 9:**
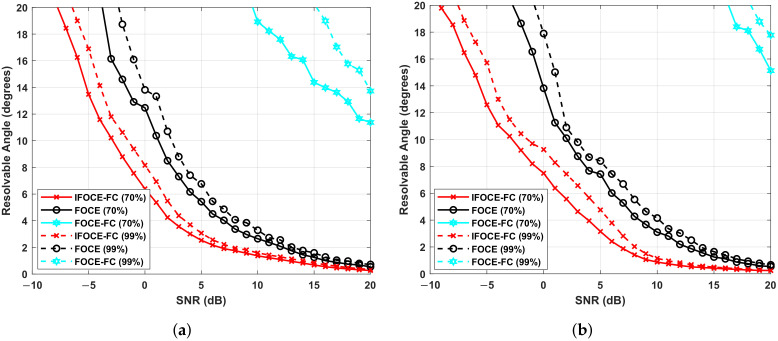
Comparison of different algorithms for the resolvable angle versus the SNR in the UCA application. (**a**) Resolvable elevation. (**b**) Resolvable azimuth.

In Figure 9a,b, we demonstrate the resolution capability of 3-D DOA estimation for UCA when $r=\frac{\lambda }{2}$. Similar criteria can be obtained by analogy to the inequality in (48). On this foundation, we demonstrate the resolvable elevation for fixed azimuth and resolvable azimuth for fixed elevation. For the former, the azimuth is set to $0^{\circ }$, whereas the elevations equal to $70^{\circ }-\frac{\beta_{\mathrm{sep}}}{2}$ and $70^{\circ }+\frac{\beta_{\mathrm{sep}}}{2}$. For the latter, the elevation is set to $70^{\circ }$, whereas the azimuths equal to $-\frac{\varphi_{\mathrm{sep}}}{2}$ and $\frac{\varphi_{\mathrm{sep}}}{2}$. Similarly, we can observe that the IFOCE-FC algorithm has better resolution performance than the others for UCA. Compared to the results of ULA, higher SNR is required for same resolvable angle and the SNR threshold increases as well. In particular, the requirement SNR for azimuth resolution is greater than the elevation resolution under same probability.

### 7.2. Estimation Accuracy for ULA

Besides the performance of resolution, we also evaluate the DOA estimation performance of the IFOCE-FC algorithm. To evaluate the estimation error of all incoming signals, the RMSE of each signal is averaged by

(50) $${\mathrm{RMSE}}_{\mathrm{ULA}}=\sqrt{\frac{1}{Q}\sum_{j=1}^{Q}\mathrm{RMSE}{\left(\theta_{j}\right)}^{2}}$$

The simulation analyzes the DOA estimation performance of the proposed algorithm and its corresponding CRLB under different SNR. We compare the performance of different algorithms, including the IFOCE-FC, the ML estimation with neighboring coupled array (MLE-NC) proposed in [37], the FOCE-FC and FOCE. The CRLB corresponding to these algorithms are labeled as CRLB IFOCE-FC, CRLB MLE-NC, CRLB FOCE-FC and CRLB FOCE, respectively. The CRLB MLE-NC and CRLB FOCE-FC can be obtained by different $H_{I}\left(j\omega_{c}\right)$ corresponding to their respective coupled structure and $\Psi_{j}=I_{M}$, whereas the CRLB FOCE can be obtained by $H_{I}\left(j\omega_{c}\right)=\Psi_{j}=I_{M}$.

In this simulation, three uncorrelated narrowband signals with equal power incident to the array from $\theta =[-5^{\circ },0^{\circ },5^{\circ }]$, which locate in the range of biologically inspired direction-finding, whereas the SNR ranges from −5 dB to 15 dB and the covariance matrix $P_{e}$ is determined according to (46).

**Figure 10:**
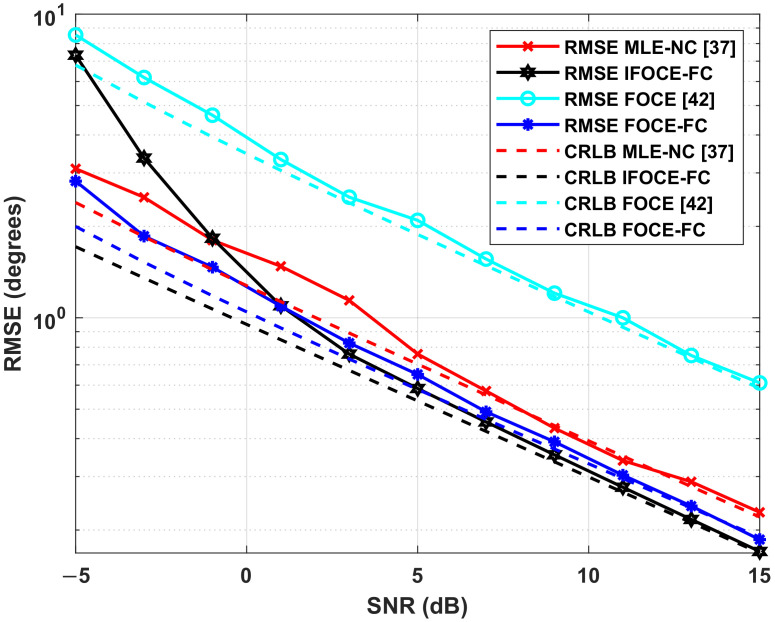
Comparison of different algorithms for the RMSE and corresponding CRLB versus the SNR under multiple signals and spatially colored noises in the ULA application.

The results in Figure 10 indicate that the proposed IFOCE-FC algorithm shows the best estimation performance when the SNR is higher than 1 dB. However, the estimation performance of the IFOCE-FC algorithm deteriorates significantly when the SNR is less than −1 dB. A reasonable interpretation for this performance deterioration is that the phase adjustment matrices $\Psi_{j}$, j=1,2,3 are obtained with large estimation error on the incident angles in each iteration, meaning that the orientation of the mainlobe cannot fall into the range of biologically inspired direction-finding. Therefore, it may sometimes be even worse than the performance without iterative scheme (FOCE-FC). Since the incident signals locate the range of biologically inspired direction-finding, the FOCE-FC algorithm has significant accuracy improvement in the given range of SNR as well. Without the iterative scheme, it shows the best performance when the SNR is less than 1 dB. Owing to the fully coupled array, both the IFOCE-FC and FOCE-FC algorithm has better performance than the MLE-NC with neighboring coupled array. Meanwhile, the FOCE algorithm without biologically inspired coupling has the worst estimation performance under the same conditions. Compared to the results in [37], the MLE-NC algorithm cannot offer satisfactory performance under low SNR. This degradation can be interpreted as the underlying correlation of the received noise being completely neglected in the MLE-NC algorithm. Thus, its object function is not suitable for the scenario with spatial colored noise. Moreover, the IFOCE-FC algorithm attains its corresponding CRLB over the SNR threshold of 3 dB.

**Figure 11:**
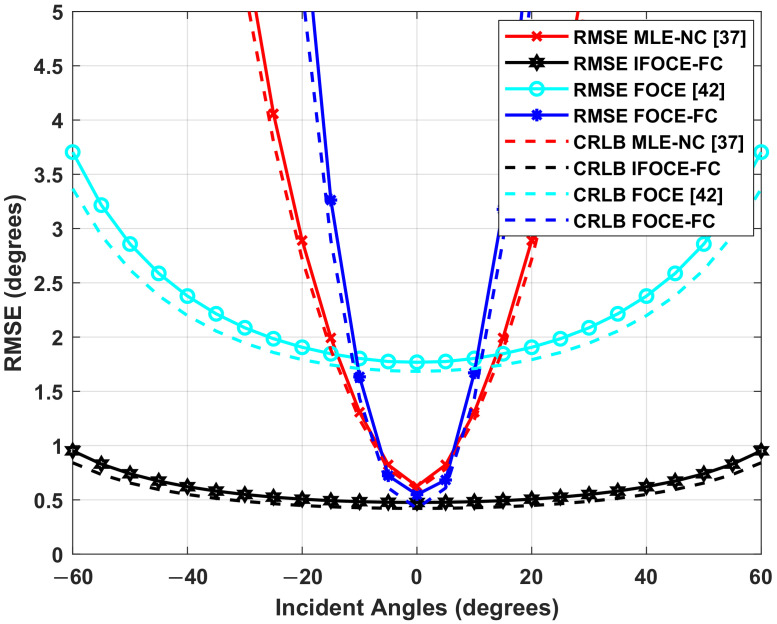
Comparison of different algorithms for the RMSE and corresponding CRLB versus the incident angle under spatially colored noises in the ULA application.

To demonstrate the effect brought by the iterative scheme, we also evaluate the RMSE of DOA estimation with respect to the incident angles. Figure 11 depicts the relation, when SNR=5dB. It can be seen that the proposed IFOCE-FC algorithm provides much better performance in the given range of incident angles than the MLE-NC and FOCE-FC algorithms. The FOCE-FC algorithm approximates the optimal performance merely within a finite range of incident angles. Meanwhile, the performance of FOCE-FC and MLE-NC algorithms deteriorate significantly when the incident angles increased. Moreover, the range of biologically inspired direction-finding for MLE-NC algorithm is larger than the FOCE-FC algorithm, which indicates its inverse proportion to the maximum accuracy improvement. It can be seen that the iterative scheme enhanced the adaptation of biologically inspired direction-finding in large incident angles, so that the needs of practical application can be satisfied.

### 7.3. Estimation Accuracy for UCA

**Figure 12:**
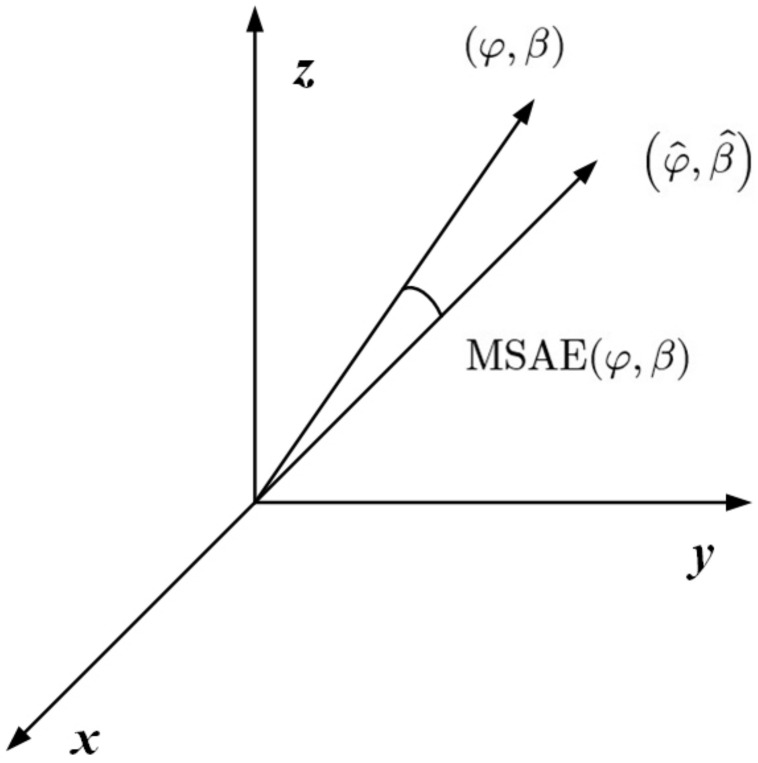
The illustration of the MSAE for 3-D DOA estimation.

In this part, we demonstrate the performance of 3-D DOA estimation for UCA. Since the performance of FOCE-FC algorithm is demonstrated above, we do not include it here to avoid redundancy. In this part, we define the mean-square angle error (MSAE) as the 3-D DOA estimation error, which is exhibited in Figure 12. It is a function with respect to the RMSE of the elevation and azimuth estimation [53]:

(51) $$\mathrm{MSAE}\left(\varphi_{j},\beta_{j}\right)=\sin^{2}\left(\beta_{j}\right)\cdot \mathrm{RMSE}\left(\varphi_{j}\right)+\mathrm{RMSE}\left(\beta_{j}\right)$$

where $\mathrm{RMSE}(\beta_{j})$ and $\mathrm{RMSE}(\varphi_{j})$ are the RMSE of the elevation and azimuth estimation corresponding to the *j*-th signal. The MSAE for all incoming signals is given by

(52) $${\mathrm{MSAE}}_{\mathrm{UCA}}=\sqrt{\frac{1}{Q}\sum_{j=1}^{Q}\mathrm{MSAE}{\left(\varphi_{j},\beta_{j}\right)}^{2}}$$

**Figure 13:**
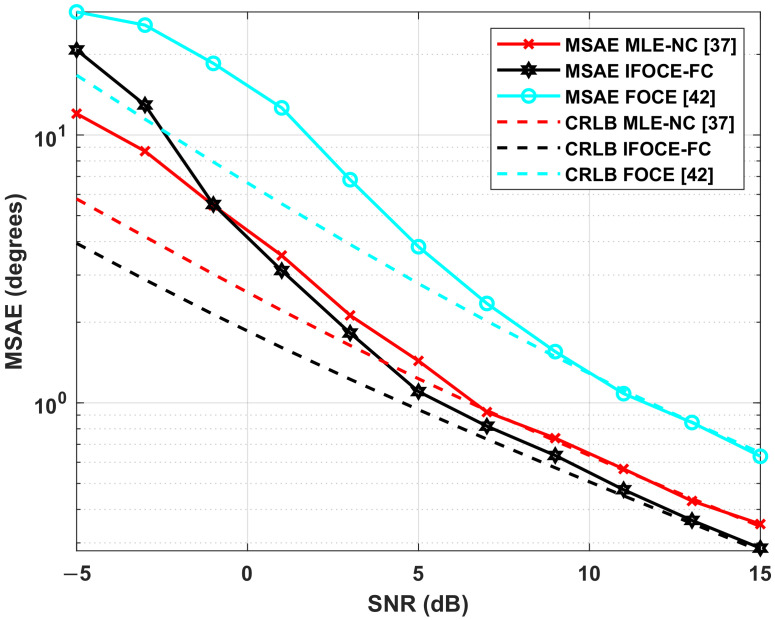
Comparison of different algorithms for the MSAE and corresponding CRLB versus the SNR under multiple signals and spatially colored noises in the UCA application.

In this simulation, the true value of elevation β and azimuth φ are $\beta =[70^{\circ },65^{\circ },60^{\circ }]$ and $\varphi =[-30^{\circ },0^{\circ },30^{\circ }]$. Figure 13 demonstrates the MSAE of 3-D DOA estimation, together with its corresponding CRLB as the function of SNR. Similar to the result for ULA, we can observe that the proposed IFOCE-FC algorithm improves the accuracy compared to the existing 3-D DOA estimation algorithms when the SNR is greater than 3 dB. We can observe that the MSAE of FOCE algorithm is 4 times that of the IFOCE-FC algorithm when the SNR is larger than 5 dB.

### 7.4. Computational Complexity Comparison

**Figure 14:**
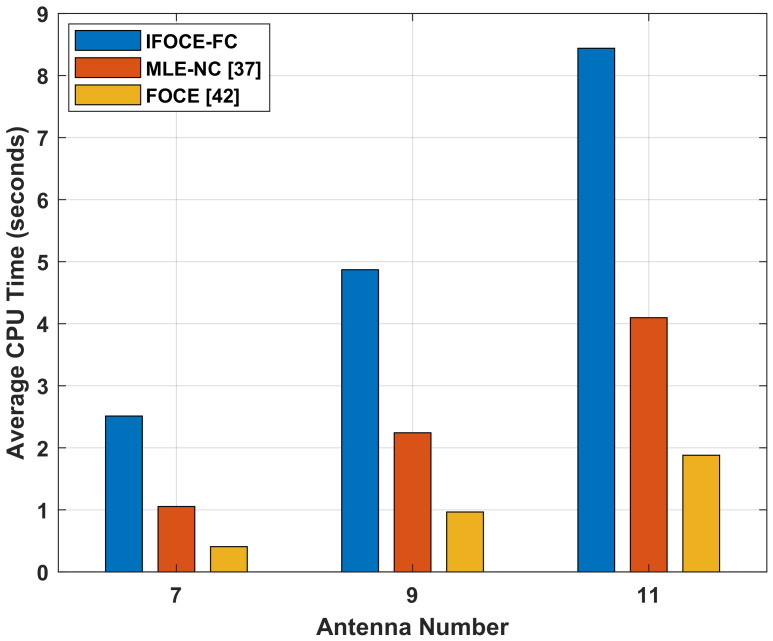
Comparison of different algorithms for the average computation time versus the number of antennas under multiple signals and spatially colored noises in the ULA application.

Finally, we compare the computational complexity of the different algorithms in the ULA application. Suppose that three narrowband signals incident to the array have SNR equal to 10 dB. The average CPU times of these algorithms for one simulation are demonstrated in Figure 14. The computer used here has dual-core 2.8 GHz CPU and 16 GB RAM. As the proposed IFOCE-FC algorithm realizes the estimation of the DOA via multi-iterations for phase adjustment, the average computational cost is most demanding. Hence, the IFOCE-FC algorithm can be used in the system without real-time requirement to improve the DOA estimation accuracy. Furthermore, the computational superiority of MLE-NC becomes secondary when taking the application on long-baseline array into consideration.

## 8. Conclusions

In this paper, biologically inspired direction-finding using the long-baseline fully coupled array is considered. First, we demonstrate the coupled structure connecting each pair of antennas and implement it as a digital filter. Then, to expand the range of biologically inspired direction-finding, we propose an iterative FOC-based DOA estimation method with fully coupled array. In this manner, the orientation of the mainlobe can be directed at the incident angle and the accuracy improvement remains at all possible incident angles. Moreover, the algorithm is insensitive to the spatial correlation of the received noises. Hence, it can estimate the DOA with unknown correlation of noises. Compared to the existing biologically inspired direction-finding algorithm and the FOC-based MUSIC algorithm, the proposed method improves both the resolution capability and DOA estimation accuracy in the presence of spatially colored noise.

The proposed algorithm merely considers the environment and amplifier noises in the mathematical model. However, in practice, the inconsistencies of amplitude and phase between the receiver channels cannot be ignored. It is necessary to modify the algorithm to overcome these above inconsistencies in practical applications. Adjustment to our algorithms should be investigated in future work.

## Data Availability

The data presented in this study are available on request from the corresponding author. The data are not publicly available due to privacy.

## Abbreviations

The following abbreviations are used in this manuscript:

DOA	Direction of arrival
ML	Maximum likelihood
MUSIC	Multiple signal classification
IFOCE	Iterative FOC-based estimation method
FC	Fully coupled
NC	Neighboring coupled
CRLB	Cramér-Rao lower bound
ULA	Uniform linear array
UCA	Uniform circular array
PDF	probability density function
SVD	Singular Value Decomposition
RMSE	Root mean-square error
MSAE	Mean-square angle error
SNR	Signal-noise ratio
BIC	Biologically inspired coupling
